# Combining Independent, Weighted *P*-Values: Achieving Computational Stability by a Systematic Expansion with Controllable Accuracy

**DOI:** 10.1371/journal.pone.0022647

**Published:** 2011-08-31

**Authors:** Gelio Alves, Yi-Kuo Yu

**Affiliations:** National Center for Biotechnology Information, National Library of Medicine, National Institutes of Health, Bethesda, Maryland, United States of America; University of East Piedmont, Italy

## Abstract

Given the expanding availability of scientific data and tools to analyze them, combining different assessments of the same piece of information has become increasingly important for social, biological, and even physical sciences. This task demands, to begin with, a method-independent standard, such as the 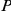-value, that can be used to assess the reliability of a piece of information. Good's formula and Fisher's method combine independent 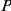-values with respectively unequal and equal weights. Both approaches may be regarded as limiting instances of a general case of combining 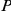-values from 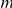 groups; 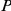-values within each group are weighted equally, while weight varies by group. When some of the weights become nearly degenerate, as cautioned by Good, numeric instability occurs in computation of the combined 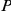-values. We deal explicitly with this difficulty by deriving a controlled expansion, in powers of differences in inverse weights, that provides both accurate statistics and stable numerics. We illustrate the utility of this systematic approach with a few examples. In addition, we also provide here an alternative derivation for the probability distribution function of the general case and show how the analytic formula obtained reduces to both Good's and Fisher's methods as special cases. A C++ program, which computes the combined 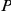-values with equal numerical stability regardless of whether weights are (nearly) degenerate or not, is available for download at our group website http://www.ncbi.nlm.nih.gov/CBBresearch/Yu/downloads/CoinedPValues.html.

## Introduction

Forming a single statistical significance out of multiple independent tests has been an important procedure in many scientific disciplines, including social psychology [1], [2], medical research [3], genetics [4], proteomics [5], genomics [6], bioinformatics [7], [8] and so on. Among the best known approaches are Fisher's method [9] and Good's formula [10]. To form a single significance assignment out of 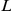 independent tail-area probabilities, Fisher's method combines these 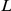 probabilities democratically while Good's formula weights every probability differently. Being able to weight more on better trusted 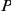-values, Good's formula is versatile. Nevertheless, it suffers from numerical instabilities when weights are nearly degenerate [10]. This paper provides an analytic formula (see eq. (33)) to properly handle nearly degenerate weights. Employing complex variable theory, we have derived this controlled expansion, in powers of differences in inverse weights, that affords for the first time both accurate statistics and stable numerics.

In addition to the scenarios covered by Fisher's method and Good's formula, one may foresee the occurrence of the following *general case* (GC): independent 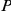-values are categorized into groups within each of which 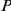-values have the same weight, while weight varies by group. The criterion for grouping can be very general, ranging from previously known attributes to differences in experimental protocols. As an example, one may wish to group data and their associated 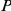-values by type of experimental instruments and assign each group a different weight. When there is only one instrument type, the GC reduces to Fisher's consideration. When there exist no replicates within each instrument type, the GC coincides with the consideration of Good.

In [10], Good also mentioned the possibility of obtaining an analytic expression for the GC, but did not provide it. Since Good's formula [10] contains, in the denominator, pairwise differences between weights, he cautiously remarked that his formula may become ill-conditioned when weights of similar magnitudes exist and thus calculations should be done by holding more decimal places. This statement has been paraphrased by numerous authors [11]–[15], and many of them have tried to seek numerically stable alternatives at the expense of using uncontrolled approximations. However, what remained elusive was a proper procedure that both provides accurate statistics and deals with nearly degenerate weights in a numerically stable manner.

The main result of this paper is an explicit formula (eq. (33)) that can properly handle nearly degenerate weights for the GC, including Good's formula of course. This derived, controlled expansion, in powers of differences in inverse weights, affords for the first time both accurate statistics and stable numerics. Employing a complex variable integral formulation, we also provide a novel derivation of the distribution function for the GC and thus become the first, in the context of combining 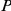-values, to make available an analytic formula for the probability distribution function for the GC.

In the statistics community, attempts to obtain an overall significance level for the results of *independent* runs of studies date back to the 1930s [9], [16]–[18], if not earlier. Nevertheless, one should note that the mathematical underpinnings of combining 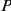-values also appear in other areas of research. For example, the equivalent of Good's formula had emerged in 1910 in the context of sequential radioactive decay [19], while the first analytic expression for Fisher's combined 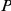-value had emerged in 1960 as a special case of the former when all the decay constants are identical [20]. After Good's work [10], Good's formula was rederived by McGill and Gibbon [21], and later on by Likes [22]. As for the GC, Fisher's method included, the mathematical equivalents appear in different areas of studies mainly under the consideration of sum of exponential/gamma variables. The distribution functions of linear combinations of exponential/gamma variables are useful in various fields. When limited to exponential variables, it results in the Erlang distribution that is often encountered in queuing theory [23]. It is also connected to the renewal theory [24] and time series problem [25], and it can be applied to model reliability [26]. The intimate connections between these seemingly different problems are not obvious at first glance. Consequently, it is not surprising that the distribution function of the GC has been rediscovered/rederived many times and that some information about it has not been widely circulated. Our literature searches show that the first explicit result (without further derivatives involved) for the distribution function for the GC was obtained by Mathai [27]. Subsequently, motivated by different contexts, Harrison [28], Amari and Mirsa [29], and Jasiulewicz and Kordecki [26] all rederived the same distribution function.

There also exist numerical approaches for combining independent 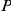-values. These typically involve inverting cumulative distribution functions. For example, Stouffer's z-methods [1], whether unweighted [30] or weighted [31], [32], require inverting the error function. Lancaster's generalization [33], [34] of Fisher's formalism also requires inverting gamma distribution function to incorporate unequal weighting for 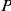-values combined. Since our main focus is on analytic approaches, we shall refrain from delving into any numerical method.

In the Methods section, we will first summarize Fisher's and Good's methods for combining 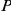-values, then present the mathematical definition of the GC. In the Results section, the subsection headed by “Derivation of 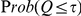
” is devoted to the derivation of the probability distribution function and cumulative probability for the GC. Since both Fisher's and Good's considerations arise as special limiting cases of the GC, we also illustrate there that our cumulative probability distribution for the GC indeed reduces to the appropriate limiting formulas upon taking appropriate parameters. In the subsection headed by “Accommodation of arbitrary weights”, we delve into our main innovative part – taming the instability caused by nearly degenerate weights – and provide a formula with controllable accuracy for combining 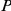-values. A few examples of using the main results are then provided in the Example subsection. This paper then concludes with the Discussion section. A C++ program CoinedPValues, which combines independent weighted P -values with equal numerical stability regardless of whether weights are (nearly) degenerate, is available for download at our group website: http://www.ncbi.nlm.nih.gov/CBBresearch/Yu/downloads/CoinedPValues.html.

## Methods

### Summary of Fisher's and Good's methods for combining 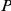-values

Assume that a piece of information is assessed by 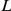 independent tests, each yielding a 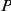-value. Each 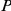-value obtained is between zero and one since, by definition, it is the probability for the experimental outcome to arise from the null model. Prior to combining these 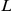 independent 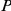-values 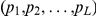 to form a single significance level, we note the following. Although for any null model 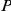-value must distribute uniformly over 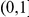, the 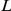 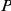-values obtained need not have their average close to 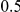. This is especially the case when the piece of information we are evaluating is not well described by the null model(s) considered.

For later convenience, let us define
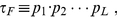
(1)

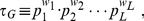
(2)where 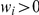 is the weight associated with the 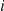
th 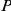-value. To form a unified significance, Fisher and Good considered respectively the stochastic quantities 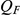 and 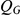, defined by
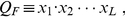
(3)

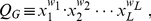
(4)where each 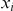 represents a random variable drawn from an uniform, independent distribution over 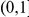. The following probabilities
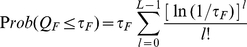
(5)

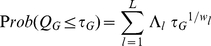
(6)provide the unified statistical significances, corresponding respectively to Fisher's and Good's considerations, from combining 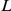 independent 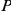-values. In eq. (6), the prefactor 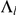 is given by
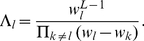
(7)Apparently, 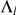 is ill-defined when the weight 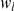 coincides with or is numerically close to any other weights 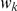. Although Fisher did not derive (5), from this point on, we shall refer to (5) as Fisher's formula and (6) as Good's formula.

### General case including Fisher's and Good's formulas

Let us divide the 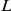 independent 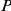-values into 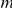 groups with 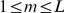. Within each group 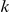, we weight the 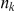 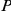-values equally; while 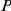-values in different groups are weighted differently. Therefore, when 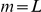 and 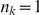 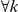, we have the Good's case; when 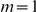 and 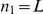, we reach Fisher's case. We will hence define the following quantities of interest
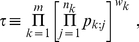
(8)

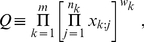
(9)where each 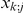 represents again a random variable drawn from an uniform, independent distribution over 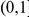. The quantity of interest 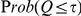, if obtained, should cover results of both Fisher and Good as the limiting cases. In the next section, we will start by deriving an exact expression for 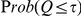 and describing how to recover the results of Fisher and Good.

## Results

### Derivation of 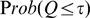


Let 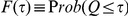, we may then write
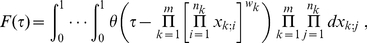
(10)where 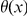 is the heaviside step function, taking value 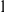 when 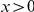 and value 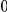 when 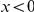. Upon taking a derivative with respect to 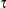, we obtain
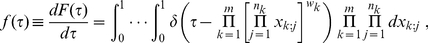
(11)where 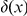 is Dirac's delta function that takes value 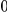 everywhere except at 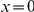 and that 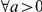, 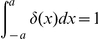.

To proceed, let us make the following change of variables
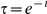


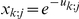
and remember that if 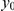 is the only root of 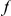 (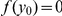)
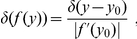
we may then rewrite (11) as
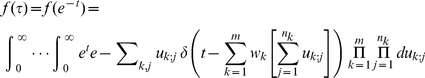
(12)

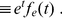
(13)Note that 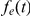 is exactly the probability density function of a weighted, linear sum of exponential variables.

By introducing the integral representation of the 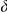 function
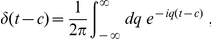
we may re-express (12) as
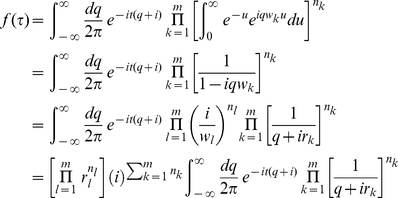
(14)

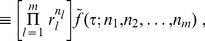
(15)where 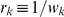 is introduced for the ease of analytic manipulation and 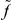 is introduced for later convenience. Since all 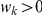, implying that all 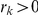, the poles of the integrand in (14) lie completely at the lower half of the 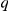-plane. Consequently, the integral of 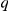 may be extended to enclose the lower half 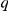-plane to result in
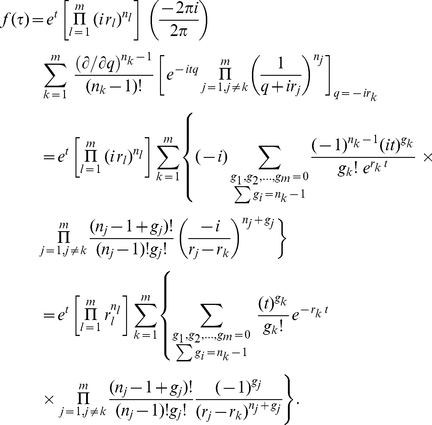
(16)Comparing eq. (16) with eqs. (12) and (13), we see that the right hand side of (16) is composed of the product of the factor 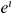 and 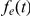 of eq. (13). In fact, the explicit expression for 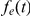, in addition to the new derivation presented here in eq. (16), was derived much earlier [27] under different context and was rediscovered/rederived multiple times [26], [28], [29] by different means. Its connection to combining 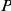-values, however, was never made explicit until now.

From (10), we know that 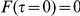, implying that
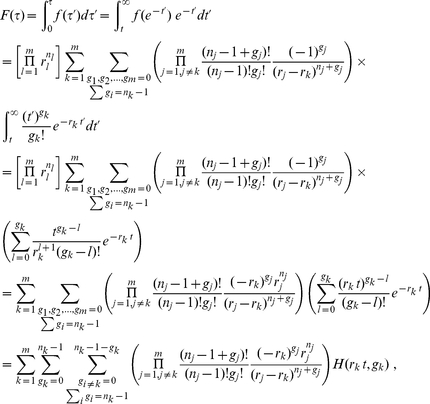
(17)where the function 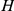 is defined as
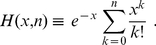
(18)Eq. (17) represents the most general formula that interpolates the scenarios considered by both Fisher and Good.

Let us take the limiting cases from (17). For Fisher's formula, one weights every 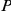-value equally, and thus corresponds to 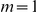 and 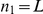. The constraint in the sum of (17) forces 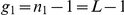. Consequently, we have (by calling 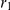 by 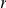 for simplicity)
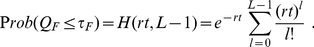
(19)Notice that regardless whatever the weight 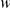 one assigns to all the 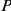-values, the final answer is independent of the weight. This is because 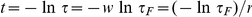 and therefore 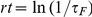. This results in
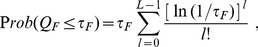
(20)exactly what one anticipates from (5). To obtain the results of Good, one simply makes 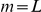 and 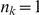 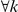, implying all 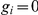. In this case, (17) becomes (with 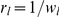, 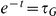 and 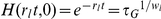)
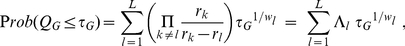
(21)reproducing exactly (6).

One may also re-express eq. (17) in a slightly different form
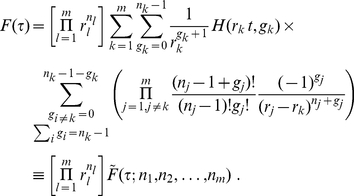
(22)Note that in the expression (22), we have isolated an overall multiplying factor and have kept explicit the 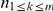 dependence for later convenience. As cautioned by Good [10] regarding Good's formula, the products of the inverse weight differences in eq. (22) may cause numerical instability in computing the combined 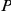-values when some of the inverse weights become nearly degenerate. To see this point, let us consider varying 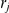 from a bit smaller than 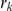 to a bit larger than 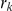. Although the change of weight 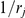 is infinitesimal, some terms in (22) do change abruptly. We will provide some numerical examples in the Example subsection.

### Accommodations of arbitrary weights

In our derivation of (20) in the previous subsection, it is explicitly shown that the final 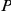-value obtained is independent of the weight 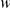 that was assigned to all the individual 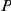-values, 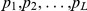. It is thus natural to ask, if one starts by weighing each 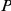-value differently, upon making the weights close to one another, will one recover Fisher's formula (5) from Good's formula (6) in the limit of degenerate weights? By continuity, the answer is expected to be affirmative. In the broader context of the GC, one would like to have a formal protocol to compute the combined 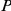-value when some of the weights become (nearly) degenerate.

In this subsection, we first illustrate the transition from Good's formula to Fisher's formula by combining two 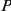-values with almost degenerate weights. We will then provide a general protocol to deal explicitly with the numerical instability caused by nearly degenerate weights. Possible occurrences of this instability were first cautioned by Good [10] and subsequently by many authors [13]–[15].

Let us consider combining 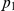 and 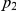 with weights 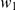 and 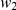 using Good's formula. One has
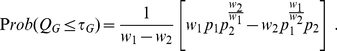
(23)Without loss of generality, one assumes 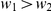 and hence writes 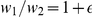 with 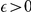. We are interested in the case when the weights get close to each other, or when 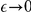. We now rewrite eq. (23) as
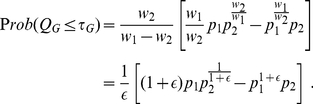
(24)In the limit of small 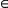, we may rewrite (24) as
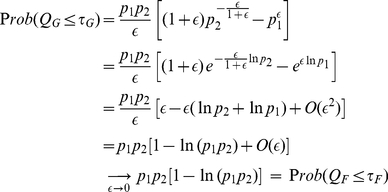
(25)Note that when the small weight difference 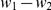 is near the machine precision of a digital computer, using formula (6) directly will inevitably introduce numerical instability caused by rounding errors.

To construct a protocol to deal with nearly degenerate weights, one first observes from eqs. (14–22) that it is the inverse weights 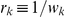 that permeate the derivation of the unified 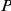-value. The closeness between weights is thus naturally defined by closeness in the inverse weights. As shown in eqs. (2) and (6), the combined 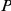-value yielded by Good's formula depends only on the pairwise ratios of the weights. Making the observation that 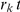 in eq. (17) only depends on the ratios 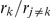, one deduces that for the GC the combined 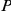-values (see (17)) also depend only on the ratios of weights, not the individual weights. We are thus free to choose any scale we wish. For simplicity, we normalize the inverse weight associated with each method by demanding the sum of inverse weights equal the total number of methods
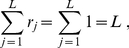
(26)where 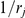 represents the weight associated method 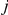 and 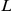 represents the total number of 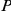-values (or methods) to be combined. For the GC described in the Methods section, 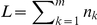. This normalization choice makes the average inverse weight of participating methods be 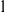.

The next step is to determine, for a given list of inverse weights and the radius for clustering, the number of clusters needed. This task may be achieved in a hierarchical manner. After normalizing the inverse weights 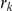 using eq. (26), one may sort the inverse weights in either ascending or descending order. For a given radius 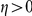, one starts to seek the pair of inverse weights that are closest but not identical, and check if their difference is smaller than the radius 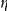. If yes, one will merge that pair of inverse weights by using their average, weighted by number of occurrences, as the new center and continue the process until every inverse weight in the list is separated by a distance farther than 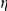. We use an example of 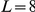 to illustrate the idea. Let the normalized inverse weights 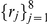 be
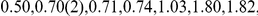
where the number 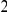 inside the pair of parentheses after 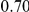 simply indicates that there are two identical inverse weights 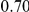 to start with. Assume that one chooses 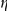, the radius for clustering, to be 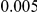. Since every pair of adjacent inverse weights are separated by more than 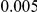, no further clustering procedures is needed and one ends up having seven effective clusters: one cluster with two identical inverse weights 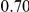, and six singletons. This corresponds to 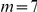, 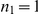, 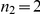, 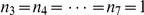.

Suppose one chooses the clustering radius 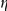 to be 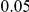. In the first step, we identify that 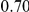 and 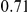 are the closest pair of inverse weights. The weighted average between them is
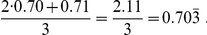
The list of inverse weights then appears as
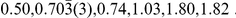
The closest pair of inverse weights is now between 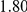 and 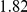, and upon merging them the list becomes
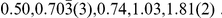
The next pair of closest inverse weights is then 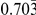 and 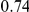. The weighted average leads to 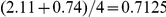. After this step, the difference between any two cluster centers is larger than 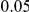. The list of inverse weights now appears as
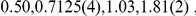
indicating that we have 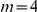 ( four clusters), with number of members being 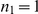, 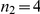, 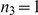 and 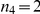. The centers of the four clusters are specified by the averaged inverse weights: 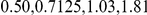.

This is a good place for us to introduce some notation. We shall denote by 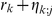 the 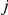
th inverse weights of cluster 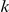, whose averaged inverse weight is 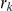. With this definition, for the example above, we have 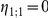, 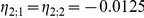, 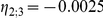, 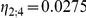, 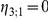, 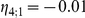, and 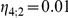.

Using the hierarchical protocol mentioned above, the number of clusters 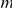, the center 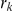 of the inverse weight of cluster 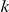, and the numbers of members 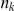 of cluster 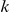 are all obtained along with 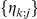 once and for all. The 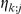, as will be shown later, constitute the key expansion parameters that yield, upon multiplying by 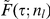 with different 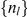, the higher order terms in our key result. We show below how this is done.

Following the derivation in the previous subsection, we obtain a probability density function very similar to (14)
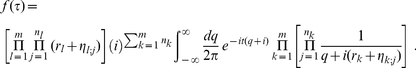
(27)


From the preceding subsection, we see that the ill-conditioned situations emerge when some weights are nearly degenerate and the source of difference in inverse weights comes from obtaining 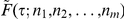 in (22) from 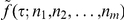 in (15). Therefore, one may leave the prefactor 
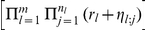
 untouched and focus on the rest of the right hand side of eq. (27). To proceed, we write
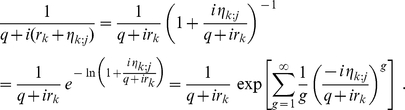
Consequently, we may write
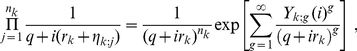
(28)where
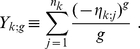
(29)The product in eq. (27) may now be formally written as
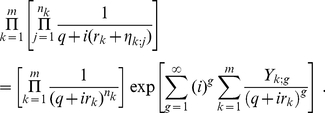
(30)


We now note a simplification by choosing 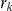 to be the average inverse weight of the 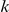
th cluster. In this case, we have 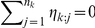 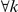. That is, 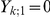 always. This allows us to write eq. (30) as
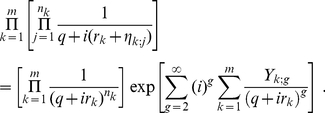
(31)


The key idea here is to Taylor expand the exponential and collect terms of equal number of 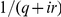. Evidently, the first correction term starts with 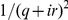. Furthermore, before the 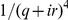 order, there is no mixing between different clusters. Below, we rewrite eq. (27) to include the first few orders of correction terms
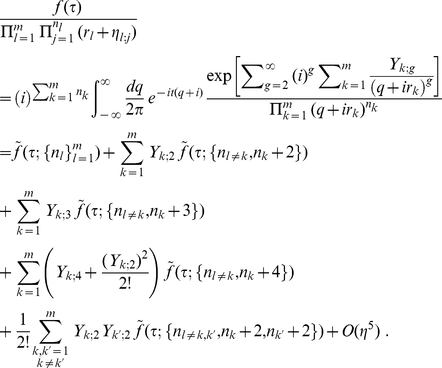
(32)This immediately leads to
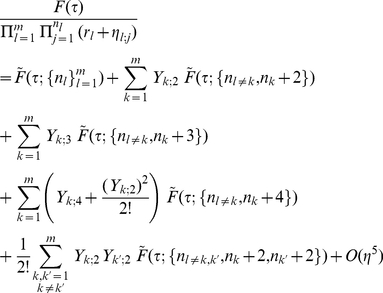
(33)


Note that when the clustering radius 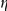 is chosen to be zero, the only clusters are from groups of *identical* weights, and all 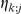 must be zero. In this case, only the first term on the right hand side of (33) exists and the result derived in the previous subsection is recovered exactly. Since all 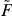 are finite positive quantities, the errors resulting from truncating the expression in eq. (33) at certain order of 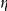 can be easily bounded. Therefore, any desired precision may be obtained via including the corresponding number of higher order terms. As the main result of the current paper, our expansion provides a systematic, numerically stable method to achieve desired accuracy in computing combined 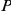-values.

### Examples

#### Example (a)

This example provides a numerical work flow to compute the 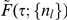 function present in eq. (22). Assuming 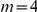, we show below how to open up the sum in eq. (22). The constraint 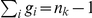 implies that one only has 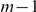 (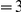 here) independent 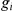
s. Once the 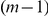 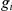
s are specified, the remaining one is determined. To simplify the exposition, let us introduce the following notation
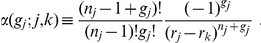
This allows one to expand the sum in (22) as
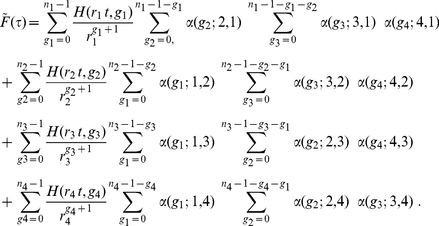
(34)


Note that in eq. (33), in the zeroth order term, the argument 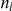 of 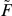 represents the number of members associated with cluster 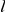. However, for higher order correction terms, the 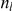
s entering 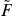 no longer carry the same meaning. Therefore, in the example shown here, one should not assume that 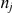 is the number of methods associated with cluster 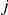.

#### Example (b)

This example illustrates the possibility of numerical instability associated with eqs. (6) and (22) when they are used to combine *P*-values with nearly equal weights. This instability arises from adding numbers with nearly identical magnitude but different signs, yielding a value containing few or no significant figures. We also show how such instabilities are resolved by using eq. (33). Consider the case of combining five 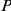-values, {0.008000257, 0.008579261, 0.0008911761, 0.006967988, 0.004973110}, weighted respectively by {0.54531152, 0.54532057, 0.54531221, 0.54531399, 0.54531776}. Using eq. (2), one obtains 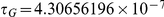. The combined 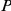-value is then obtained as the probability of attaining a random variable 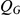, defined in eq. (4), such that it is less than or equal to 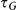.

Combining 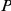-values using eq. (6) gives
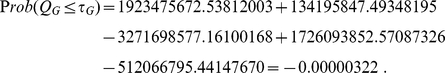
When one uses equation (22), 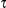 takes the value of 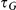 and the random variable 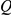 is simply 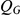, and the combined 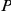-value becomes
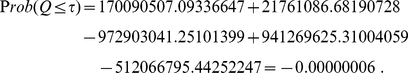
Apparently, probability cannot be negative. The negative values shown above illustrate how eqs. (6) and (22) may lead to cancellation of numbers of comparable magnitude thus may yield meaningless values when the weights are nearly degenerate. This numerical instability is removed by applying equation (33), which combines weighted 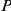-values using a controlled expansion and yields, for this example,
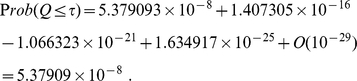



#### Example (c)

One natural question to ask is how well does eq. (33) work when one chooses a larger clustering radius and group weights that are clearly distinguishable into a few clusters? To consider this case, let us use the five 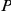-values from example (b) but with weights chosen differently. Let us assume that the inverse weights (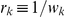) associated with these five 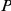-values are 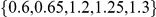. For this case, 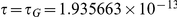. Combining 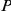-value using formulas (6) yields
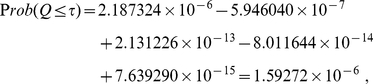
while combining 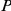-values using (22) yields identical results
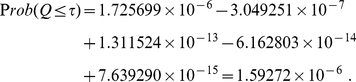



When one uses 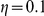 as the clustering radius, one obtains two clusters: one with average inverse weight 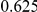 and the other with average inverse weight 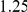. If one then uses eq. (33) to combine 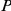-values, one attains the following results
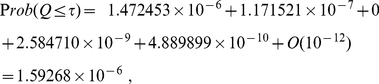
(35)which contains no sign alternation and agrees well with the results from both (6) and (22). This illustrates the robustness of eq. (33) in combining 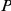-values. Note that the third term on the right hand side of (35) is zero. This is because the multiplying factor 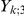 is zero for both clusters. In general, 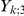 measures the skewness of inverse weights associated with cluster 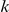 and for our case here both clusters of inverse weights are perfectly symmetrical with respect to their centers, leading to zero skewness. If the inverse weights of cluster 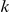 distribute perfectly symmetrically with respect to its center, it is evident from eq. (29) that 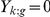 for odd 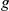.

Evidently if one chooses a large clustering radius 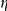 and then uses eq. (33) to combine 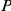-values, many higher order terms in the expansion will be required to achieve high accuracy in the final combined 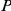-value.

## Discussion

Although the expression (17) provides access to exact statistics for a broader domain of problems and our expansion formula (33) provides accurate and stable statistics even when nearly degenerate weights are present, there remain a few unanswered questions that should be addressed by the community in the near future. For example, even though we can accommodate any reasonable 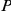-value weighting, thanks to (33), the more difficult question is how does one choose the right set of weights when combining statistical significance [35]–[39]. The weights chosen should reflect how much one wishes to trust various obtained 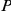-values. Ideally, a fully systematic method should also provide a metric for choosing appropriate weights. How to obtain the best set of weights remains an open problem and definitely deserves further investigations.

Another limitation of (17) and (33), and consequently of Fisher's and Good's formulas, is that one must assume the 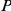-values to be combined are independent. In real applications, it is foreseeable that 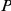-values reported by various methods may exhibit non-negligible correlations. How to obtain the correlation [40]–[42] and how to properly incorporate 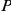-value correlations [15], [43], [44] while combining 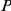-values are challenging problems that we hope to address in the near future.
